# A flow map for core/shell microdroplet formation in the co-flow Microchannel using ternary phase-field numerical model

**DOI:** 10.1038/s41598-022-26648-3

**Published:** 2022-12-20

**Authors:** Saeed Ghasemzade Bariki, Salman Movahedirad

**Affiliations:** School of Chemical Engineering, Iran University of Science and Technology, Tehran, 16846-13114 Iran

**Keywords:** Chemical engineering, Computational science, Software, Flow chemistry

## Abstract

Core/shell microdroplets formation with uniform size is investigated numerically in the co-flow microchannel. The interface and volume fraction contour between three immiscible fluids are captured using a ternary phase-field model. Previous research has shown that the effective parameters of microdroplet size are the physical properties and velocity of the three phases. By adjusting these variables, five main flow patterns are observed in numerical simulations. A core/shell dripping/slug regime is observed when the inertia of the continuous phase breaks the flow of the core and shell phases and makes a droplet. In the slug regime, the continuous phase has less inertia, and the droplets that form are surrounded by the channel walls, while in the dripping regime, the shell phase fluid is surrounded by the continuous phase. An increase in continuous-fluid or shell-fluid flow rate leads to dripping to a jetting transition. When three immiscible liquids flow continuously and parallel to one another without dispersing, this is known as laminar flow. In the tubing regime, the core phase flows continuously in the channel's central region, the shell phase flows in the annulus formed by the core phase's central region, and the continuous phase flows between the shell phase fluid and channel walls. In order to discriminate between the aforementioned flow patterns using Weber and Capillary numbers and establish regime transition criteria based on these two dimensionless variables, a flow regime map is provided. Finally, a correlation for shell thickness using shell-to-core phase velocity ratio and conducting 51 CFD simulations was proposed.

## Introduction

Aqueous three-phase flows, particularly the movement of a single core/shell micro-droplet through the third phase as a continuous phase^1^, are useful in a wide range of industrial and medical applications, including efficient heat and mass transfer^2^, nuclear safety study^3^, efficient recovery techniques^4^, tissue engineering^5^, coating technology^6^, and drug delivery systems^7^. Due to the potential of core–shell structures in areas such as drug delivery, treatment with biomedical imaging, and tumor therapy, they have become important over the past decade^8^.

A combination of three-phase flows with microfluidic technology has been widely investigated in recent decades in order to provide precise control and continuous operation^9,10^. The miniaturization of synthesis systems provides new possibilities for improved chemical synthesis, as well as a platform of biological and medical applications^11^. Core–shell microdroplets (CSMs) formation in microfluidic devices has a number of advantages: (1) improved processing precision and efficiency, (2) design flexibility for a multi-step platform, (3) quick turnaround results for fine tuning properties of shaped droplets, (4) cost savings from reduced raw material and reagent consumption, and (5) using significantly fewer potentially harmful chemicals and reagents allows for safer operations and a reduced impact on the environment^12^.

For the formation of core–shell microdroplets, the double emulsion approach is widely used^13^. In addition to other multistep and sophisticated operations, the synthesis process includes solvent evaporation, emulsion, purification, and sonication of the produced droplets^14^. The produced droplets also had modest recovery rates^15^, narrow size distributions^16^, and complex microstructures^17^. Microfluidic technologies are used to control the formation and particle size due to the difficulty of fluid management in bulk methods^18^. Solubility^19^, stability, reactivation using visual stimuli^20^, narrow size distribution, core and shell processing, and autonomic capabilities are the most essential aspects of CSMs as a building block model for functional materials^21^. Many distinct types of core–shell materials, such as single or multiple materials in plane, core/shell or composite, can be classed as core–shell materials^22^. In general, core/shell structures are defined as having an internal matter and an exterior layer material^23^.

The characteristics of core–shell drug-carrier particles are well-known^24–26^. Li et al. developed a novel microchannel device and used it for the preparation of silica-silica core–shell microspheres by combining with a Dextran/Poly (ethylene glycol) diacrylate (DEX/PEGDA) with different sizes of cores, thicknesses of shells and flow rate ratios of DEX/silica and PEGDA/silica aqueous solutions^27^. Knauer et al. synthesized colloidal dispersions of noble metal core/shell and multi-shell nanoparticles in aqueous solutions in the presence of cetyltrimethylammonium bromide (CTAB) with a two-step micro continuous flow^28^. In another study, a high-throughput screening platform using a centrifugal microfluidic device for producing combinatorial tri-metallic catalysts was proposed, where the Pd-nanocube served as a core and the Au and Pt atoms formed a shell^29^. Sun et al., investigated tunable rigidity of (polymeric core)-(lipid shell) nanoparticles in different concentration for regulated cellular uptake^30^. A continuous two-step glass-capillary microfluidic technology to build a multistage oral delivery system is presented in a study by Costa et al. Insulin is encapsulated in liposomes that have been coated with chitosan to improve mucoadhesion^31^. The enteric polymer encapsulation provides protection from the severe digestive environment. In fact, core–shell particles attract a lot of attention since they feature a mix of superior properties that the separate components don't have. The structures could combine core and shell properties and characteristics^32^. These particles were created to deliver a regulated dose of medication to a specific spot. So, the negative consequences would be reduced^33^. As a result of the advantages of core/shell structures, new methods and ideas for their manufacturing have emerged. Table 1 shows some examples of core–shell nanoparticles, their bases, and their applications.

**Table 1:** Example of core–shell structures, their base, and their application.

Core	Shell	Base	Application	References
Urea granules	Natural Oils as 1st shell, poly acrylic acid (PAAc) hydrogels as 2st shell	Polymer	Excellent water absorption in soil and slow-release behavior of urea Safe applications in farming fields	^34^
Chitosan 1%, clay, and KNO_3_	Chitosan 2%	Polymer	Development of Enhanced efficiency fertilizers (EEF) for the released nutrient and biodegradation in the soil to ensure material harmlessness	^35^
Urea granules	Zn capped with binary agents: N-acetyl cysteine (NAC) and sodium salicylate (SAL)	Metal and metal oxide	Strategies to streamline fertilization regimes are warranted due to the size-induced possibility of drifting, segregation, or transformation	^36^
Raoultella planticola (Rs-2)	Sodium bentonite and alginate (NaAlg) composites	Metal and metal oxide	Efficient development of slow-release bio-fertilizer formulations and minimization of production costs	^37^
Urea granules	Poly(3-hydroxybutyrate) (PHB) and its nano-composites (PHB/MMT and PHB/OMMT)	Polymer	Drugs, pesticides, fertilizers, and proteins releasing Enhanced of soil biodegradability and degradation rates	^38^
Candelilla wax	Phosphorus fertilizer	Nature solutions	Improvement of crop yields and enhanced of slow-release and biodegradability	^39^
Dextran	Poly (ethylene glycol) diacrylate	Silica	High stability and much easier recovery of core–shell microspheres catalyst	^27^
Au	Ag (single and multi-shell)	Metal and metal oxide	Future sensing applications, labeling in bio-analytics or nonlinear optical devices, high internal segment mixing efficiency	^28^
Pd-nanocube	Au and Pt atoms	Metal and metal oxide	Carbon dioxide reduction, water splitting, formic acid oxidation, and direct synthesis of hydrogen peroxide due to their specific electronic structure and large surface to volume ratio	^29^
Poly(lactic-co glycolic acid) (PLGA)	Lipid	Polymer	Controlled interactions between nano-materials and cells, novel therapeutic applications and the construction of bio-mimetic models such as the blood capillary, and drug delivery	^30^
Insulin	Liposomes/chitosan	Polymer	Oral drug delivery	^31^
Pd–Ag	Mesoporous silica-based nanoparticle	Silica	Photo- and pH-triggered release of anticancer drugs	^40^
Oxidized sodium alginate	Chitosan	Polymer	Development of a colon-specific delivery	^41^
Ag	Poly(N-isopropylacrylamide-co-acrylic acid)	Metal and metal oxide	pH-regulated drug delivery	^42^
Polyethylene glycol	Mesoporous silica-based nanoparticle	Silica	Targeted inhibition of notch signaling in breast cancer	^43^
Ferrite impregnated acrylonitrile	Acrylamide	Polymer	Targeted drug delivery	^44^

Extraction, polymerization, nitration, and pharmaceutical chemistry are just a few of the many fields where liquid–liquid systems are significant. Few researchers have examined liquid–liquid flow patterns and associated hydrodynamics in the literature^45–49^. Microfluidic devices often have two or more microchannels to allow for the entry of both dispersed and continuous liquid phases. The microfluidic device geometry determines where the channels converge, and the shape of the resulting junction plays a role in defining the local flow fields that distort the interface between the two fluids^50^.

Depending on the geometry of the cross-shaped junction and the microchannels, the flow rates of the two/three phases, and the characteristics of the two/three phases, a variety of flow patterns may appear. The transitions in flow patterns for a three-phase liquid–liquid–liquid flow in a microchannel are defined by the interfacial tension, viscous shear force, and liquid inertia, the relative magnitudes of which depend on the channel geometry, flow rates, and physical properties of the core, shell, and continuous phases. Core, shell, and continuous phases, as well as their associated flow structures, are all determined by the wetting qualities of the liquids with regard to the microchannel walls. Researchers observed and mapped distinct flow patterns in microchannels with varying inlet junctions using dispersed and continuous phase velocities or dimensionless numbers such as Capillary numbers for two-phase flow^51–53^. Specifically, Cubaud and Mason have observed threading, jetting, dripping, tubing, and viscous displacement for microfluidic cross-shaped junctions^54^. Since dimensional parameters in flow pattern maps are not as customizable as dimensionless numbers, dimensioned maps are not as widely applicable.

Several factors, including those mentioned above, determine flow patterns. Forces that predominate in flow patterns are the basis for these elements. Normally, transitions in flow pattern were the result of forces such as shear, inertia, and interfacial tension. Typically, flow pattern maps are based on the surface velocities of the phases or on dimensionless numbers^55^. In this work, dimensionless numbers are used to illustrate flow patterns. Consequently, in studies of liquid–liquid–liquid flow patterns, dimensionless analysis provides an efficient way for developing general flow pattern maps.

Experimentally examining three-phase flow is not usually adequate to gain a thorough understanding of the associated flow phenomena. In addition, measuring microstructures is a challenging technique. Therefore, computational fluid dynamics (CFD) has developed as an alternate method for investigating ternary flow in microchannels^56^. In order to perform a direct comparison between numerical and experimental results, the core/shell microdroplet size must match that of the experimental data. This can be accomplished by simulating the core/shell microdroplet formation configuration used in the experiment. Such modeling necessitates computationally demanding, unsteady calculations.

Droplet formation in microchannels has been extensively studied for two-phase flow^57–59^, but much less so for three-phase flow using the phase field simulation method. Wang et al. investigated coaxial electrohydrodynamic jet (CE-jet) printing. Their work proposes a novel simulation based on phase field, a precise fluid dynamics methodology. The study examines the effects of applied voltage, needle-substrate distance, dynamic viscosity, relative permittivity, needle size, and flow rate on CE-Jet morphologies^60^. In another study, a rising air bubble passes through a stratified horizontal interface between two Newtonian liquids. The interface between three immiscible fluids was modeled using a ternary phase-field model^61^.

Nearly all of the studies that came before this one relied on experimental study and extensively explored the effect of numerous parameters (such as the physical properties of three phases on various aspects of a core/shell microdroplets formation). However, few or no works conduct a comprehensive CFD investigation of the core/shell microdroplet generation, including the various regimes that can occur and the transition conditions. Due to the limits of an experimental investigation, a non-dimensional flow map was proposed that can cover all possible regimes. The goal of this study is to discover and characterize the flow regimes that follow microdroplets passing via a liquid–liquid interface. To determine the regimes, numerical simulations are performed using the phase-field model. The phase-field approach has a lot of benefits that VOF and other numerical methods don't have. This physics may automatically keep an interface's sharpness without breaking continuity, preserve an interface's structure as it evolves, and eliminate the need for an interface's reconstruction or re-initialization. In order to plot the flow regime map and determine the transition criteria between regimes, it is necessary to classify the regimes and determine the relevant dimensionless numbers.

## Modeling

The purpose of this simulation is to form a core–shell microdroplet in the center of the co-flow microchannel surrounded by the continuous phase by controlling the flow rates. In the following sections, the governing equations, simulation conditions, solution geometry, and mesh independency are presented in detail.

### Governing equations

A ternary phase-field model is used to trace the boundaries between three immiscible fluid phases, or phases 1, 2, and 3. The core, shell, and surrounding liquid are referred to as phases 1, 2, and 3, respectively. The measure of concentration of each phase is represented by a phase-field variable $(\phi$) that can take on values between 0 and 1. Phase 1's phase-field variable, for example, is 1 when just this phase is involved and 0 when no phase 1 is present. Since the fluids are immiscible, a shift in the phase-field variable indicates the presence of an interface. When two adjacent phases have phase-field variables that deviate from their limiting values, this boundary is known as the separation interface. For each point in time, the following condition must necessarily be the case for the phase-field variables^62^:

1 $$\sum_{i=1}^{3}{\phi }_{i}=1$$

Since it is assumed that the density of each phase remains constant, the phase-field variable represents the volume fraction of each phase.

Each phase's generalized chemical potential ${\eta }_{i}$ introduces a new dependent variable into the ternary phase-field, transforming it from a fourth-order partial differential equation (PDE) into two second-order PDEs^63^:

2 $$\frac{\partial {\phi }_{i}}{\partial t}+\nabla .\left({\varvec{u}}{\phi }_{i}\right)=\nabla .\left(\frac{{M}_{o}}{{\Sigma }_{j}}\nabla {\eta }_{i}\right)$$

3 $${\eta }_{i}=\frac{4{\Sigma }_{T}}{\varepsilon }\sum_{j\ne i}\left(\frac{1}{{\Sigma }_{j}}\left({\partial }_{i}F\left({\varvec{\phi}}\right)-{\partial }_{j}F\left({\varvec{\phi}}\right)\right)\right)-\frac{3}{4}\varepsilon \sum i{\nabla }^{2}{\phi }_{i}$$

where ${\varvec{u}}$ is the fluid velocity vector.

The diffusion coefficient ${M}_{o}$, also known as mobility, defines the time scale of diffusion^61^:

4 $${M}_{o}={M}_{const}({\phi }_{1}^{2}{\phi }_{2}^{2}+{\phi }_{1}^{2}{\phi }_{3}^{2}+{\phi }_{2}^{2}{\phi }_{3}^{2})$$

where ${M}_{const}=2\times {10}^{-10}{\mathrm{m}}^{2}/\mathrm{s}$. While it needs to be big enough to keep the interfacial thickness constant, it can't be too big or convective transport will be stifled. The mobility is zero in the pure phases. $\varepsilon$ can be viewed as the interface's characteristic size; it is a parameter that determines the interface's thickness and follows the same pattern as the elements in fluid–fluid interface domains.

The capillary parameter ${\Sigma }_{i}$ is defined in the following for each phase^61^:

5 $${\Sigma }_{i}={\sigma }_{ij}+{\sigma }_{ik}-{\sigma }_{jk}$$

The surface tension ($\mathrm{N}/\mathrm{m}$) of the interface between phases i and j is denoted by ${\sigma }_{ij}$. After that, the coefficient ${\Sigma }_{T}$ is defined as follows^61^:

6 $$\frac{3}{{\Sigma }_{T}}=\frac{1}{{\Sigma }_{1}}+\frac{1}{{\Sigma }_{2}}+\frac{1}{{\Sigma }_{3}}$$

To finalize the model, the phase-field physics is coupled with the Navier–Stokes equations for laminar flow. The interfaces are consistent with the principles of conservation of mass and momentum^64^:

7 $$\frac{\partial \rho }{\partial t}+\nabla .\left(\rho {\varvec{u}}\right)=0$$

8 $$\rho \frac{\partial {\varvec{u}}}{\partial t}+\left(\rho {\varvec{u}}.\nabla \right){\varvec{u}}=-\nabla p+\nabla .\left(\mu \left(\nabla {\varvec{u}}+\nabla {{\varvec{u}}}^{T}\right)\right)+\rho {\varvec{g}}+{{\varvec{F}}}_{{\varvec{s}}{\varvec{t}}}$$

where $g$ is the gravitational acceleration (m/s^2^) and *p* is the static pressure (Pa). The fluid mixture's density and viscosity are defined as follows^61^:

9 $$\rho =\sum_{i=1}^{3}{{\rho }_{i}\phi }_{i}=1$$

10 $$\mu =\sum_{i=1}^{3}{{\mu }_{i}\phi }_{i}=1$$

Because all fluids are Newtonian and incompressible, each pure phase's density and viscosity are considered to be constant.

The surface tension force is introduced to the Navier–Stokes equations as a body force by multiplying the phase's chemical potentials by the gradient of the appropriate phase-field variable^61^:

11 $${{\varvec{F}}}_{{\varvec{s}}{\varvec{t}}}=\sum_{i=1}^{3}{{\eta }_{i}\nabla \phi }_{i}$$

### Solution method

To simulate the problem, laminar flow and phase-field (three-phase flow) physics were coupled. The finite volume method can be used to discretize and solve governing equations numerically. By assigning an initial guess to the phase-field variable ($\phi$) and solving Eq. (2), generalized chemical potential, mobility and capillary parameters, phase’s physical properties, and interfacial tension force are calculated and introduced as the input parameters to the Navier–Stokes equation. Then, by calculating the velocity and pressure field, a new value of $\phi$ is obtained in the next time step, and this procedure continues until convergence.

### Geometry and boundary condition

**Figure 1:**
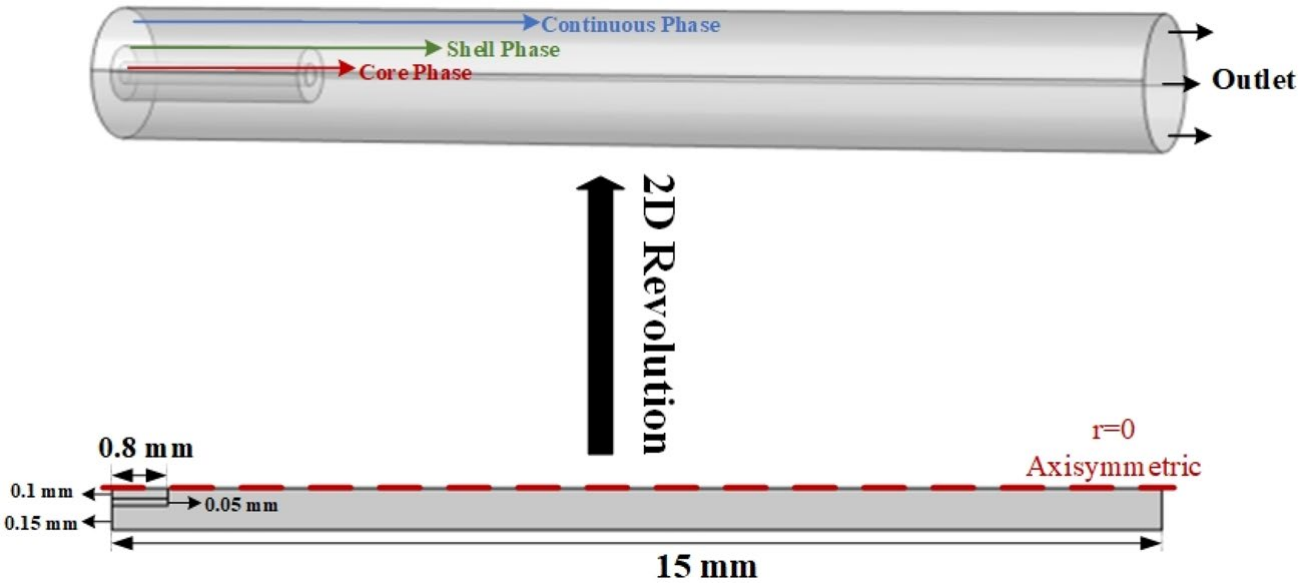
Geometry of the microchannel.

In the three-phase microfluidic method, the core, shell, and continuous phases generally enter the micro-channel from three separate inlets. The geometry of the three-phase junction strongly affects the droplet size and helps to define the local flow fields that show the deformation of the interface between the three phases. For this purpose, co-flow geometry with an inner diameter of 0.6 mm was used. For the core and shell phases respectively, a circular channel with an inner radius of 0.2 mm and an outer radius of 0.3 mm was set in the channel’s center, as can be seen in Fig. 1. It should be noted that the simulation of the micro-channel was done in two dimensions (2D) with axial symmetry.

Numerical simulations have traditionally been used to resolve the flow's small spatial and temporal scales, allowing access too many details of the flow that would be impossible to obtain experimentally. Existing fundamental analyses, however, have primarily focused on circular channels that can be modeled with 2D axisymmetric geometries. This option can be used if our model's geometry and the loads and constraints you intend to apply to it are symmetric about an axis, such as cylindrical and conical structures like tanks, flanges, or certain clamps. 2D axisymmetric models are a slice of the actual 3D model that would become the original 3D structure if rotated around the Y-axis of the reference Cartesian coordinate system or the r-axis of the reference cylindrical coordinate system.

In 2D axisymmetric models:

- All included geometry must be in the r–z plane of the cylindrical coordinate system chosen as the model's reference coordinate system.
- All of the geometry must be located in the z > = 0 region of the r–z plane.
- The r–z plane is where stresses and deformations must be specified.
- Symmetric shape of the channel and flow pattern.

If the chosen model meets these requirements, a section of the structural system can be represented as a 2D axisymmetric model.

Axisymmetric flow was simulated by adjusting several input parameters to calculate the volume fraction of the phases. The accuracy of the simulation is 97.1% since there is a 2.9% difference between the diameter of the core/shell microdroplet derived from the simulation and the experimental results. Although axisymmetric models are uncommon, they can be a useful alternative to 3D modeling, which is a time-consuming effort. It should be noted that this technique can only lead to accurate results if its assumptions are met. In this investigation, by comparing axisymmetric simulation with 3D modeling, it was found that the axisymmetric technique was ten times faster than compute modeling. Axisymmetric models may help speed up the process of figuring out the right level of grid resolution for 3D models and estimating aquifer parameters from field experiments.

As can be seen in Fig. 1, the microchannel has three inlets: one for the core phase, one for the shell phase, and one for the continuous phase. The inlet velocity condition is chosen for all of these inlets. The pressure outlet is a boundary condition at the micro-channel’s output. A wetted wall condition with a contact angle of 135 degrees is used for the microchannel outer walls and the core/shell walls. The inner interface is the boundary condition on the output side of the core/shell phase. Table 2 provides a summary of the co-flow device's boundary conditions and their associated values.

**Table 2:** Boundary conditions for considered geometry.

Co-flow device	Boundary condition	Value or range
Core phase inlet	Velocity inlet	${u}_{Core}=9.09\times {10}^{-5}-1.03\times {10}^{-1}\mathrm{m}/\mathrm{s}$
Shell phase input	Velocity inlet	${u}_{Shell}=3.41\times {10}^{-4}-3.69\times {10}^{-1} \mathrm{m}/\mathrm{s}$
Continuous phase input	Velocity inlet	${u}_{C}=1.42\times {10}^{-3}-2.21\times {10}^{-1} \mathrm{m}/\mathrm{s}$
Microchannel output	Pressure outlet	${P}_{Out}=0 \mathrm{bar}$
The inner and outer wall of the microchannel	Wetted wall	${\theta }_{w}=2.5\pi /4-3\pi /4$
Dispersed phase output	Interior interface	–

### Simulation conditions

Designed and extracted geometry was fed to the CFD simulator after mesh generation. Inlet velocities have been set for the core, shell, and surrounding phases. The equations, which were based on a finite element discretization and a backward differentiation formula (BDF) for the time step scheme, were solved using the time-dependent multifrontal massively parallel sparse direct solver (MUMPS). The finite element method (FEM) offers good flexibility and accuracy for time-dependent simulations while making complicated geometrical and asymmetrical shapes easier to model. The total computation time on an AMD Quad Core 64-bit 3.1 GHz processor was around 420 min.

As mentioned before, the problem-solving method was based on fluid incompressibility and unsteady-state conditions. Due to the low Reynolds number (*Re*), the selected flow model is laminar flow in the simulations. The surface tension between each two phases was considered constant. Then, using mixing rules, the phase’s physical properties are obtained and introduced to the simulation as input parameters. The phase-field method has been selected to model three-phase flows. The time step under consideration is 10^–4^ s, and simulations were performed in 1000-time-steps.

### Mesh independency

The mesh size has a direct effect on the simulation results. In order to capture the formation of a core–shell microdroplet, a very fine mesh was considered for the three-phase collision site. Normal grids can be used in the rest of the microchannel domains to optimize and minimize computational time. After considering droplet motion, form evolution over time, and the geometric complexity of the microdroplets interaction with the liquid–liquid interface, a non-structured mesh with triangular elements was used for discretization.

As can be inferred from Table 3, the computational domain was discretized using 349,763 triangular mesh elements. The solution's independence from the mesh size was verified by performing calculations on a mesh with 1,430,567 elements. Over the entire domain, the dissimilarities between the solutions for the two meshes mentioned above were found to be less than 2%. Therefore, the mesh density report can be adjusted according to Table 3.

**Table 3:** Mesh density report for determining core–shell microdroplet size and validation in the co-flow device.

No. of meshes	Mesh geometry	Maximum element size	Microdroplet size (μm)/ Experimental^27^	Microdroplet size (μm)/ Simulation	Error (%)	Computational Time (hour)
6564		0.1	300	360	16.67	10
14,307		0.04	300	338	11.24	16
222,778		0.01	300	321	6.54	26
349,763		0.008	300	309	2.91	31
1,430,567		0.004	300	303	0.99	52

As shown in Table 3, for 349,763 meshes, it takes 31 h to solve the problem with a time step of 10^–4^ s approximately. For this number of meshes, the problem is almost optimized and there is no need to add meshes to solve the problem more precisely. According to Table 3, the error percentage of the problem with 349,763 meshes almost loses its incremental trend and becomes reliable. So, this number of meshes can be chosen as the best mesh for simulating the problem.

### Theoretical background and dimensionless groups

**Figure 2:**
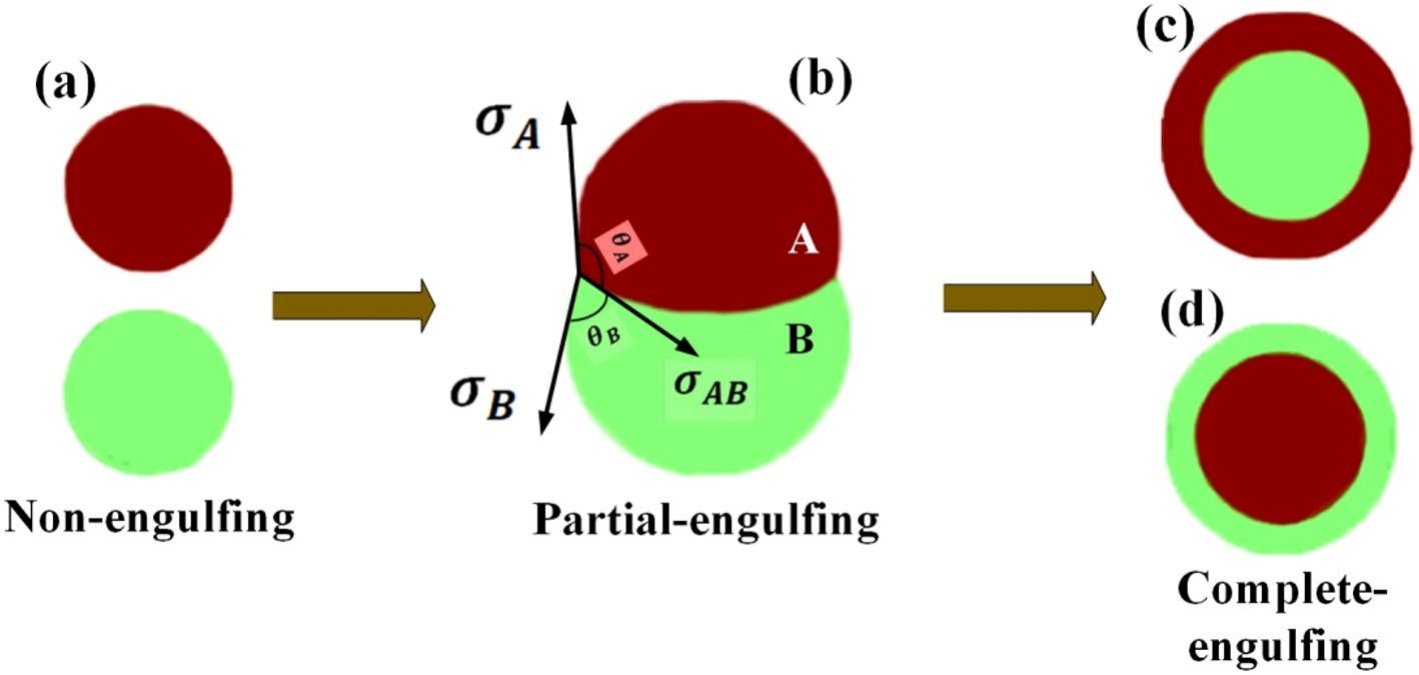
The contact condition and the effect of interfacial tension of the core/shell droplet.

The interfacial tension has a significant effect on the morphology of microdroplets. Figure 2 illustrates a model schematic of a core/shell droplet made up of two incompatible phases A and B. phase A and the interfacial tension of continuous phase is represented by ${\sigma }_{A}$, and phase B and the interfacial tension of continuous phase is indicated by ${\sigma }_{B}$. The three interfacial forces acting along the interfacial are in equilibrium, with ${\sigma }_{AB}$ being the interfacial tension between phase A and phase B, ${\theta }_{A}$ being the angle between ${\sigma }_{A}$ and ${\sigma }_{AB}$, and ${\theta }_{B}$ being the angle between ${\sigma }_{B}$, and ${\sigma }_{AB}$. Three primary morphologies are represented in Fig. 2:

1. *Non-engulfing* Droplets of phase A are kept separate from droplets of phase B when ${\sigma }_{A}$, ${\sigma }_{B}$, and ${\sigma }_{AB}$ fulfill formula (12), as depicted in Fig. 2a.
  12 $${\sigma }_{AB}>{\sigma }_{A}+{\sigma }_{B}$$
2. *Complete engulfing* The phase A droplet completely engulfs the phase B droplet, creating a droplet with the B@A core–shell structure, as seen in Fig. 2c, when ${\sigma }_{A}$, ${\sigma }_{B}$, and ${\sigma }_{AB}$ fulfill the formula (13).
  13 $${\sigma }_{B}>{\sigma }_{A}+{\sigma }_{AB}$$
  The phase B droplet completely engulfs the phase A droplet, creating a droplet with the A@B core–shell structure, as seen in Fig. 2d, when ${\sigma }_{A}$, ${\sigma }_{B}$, and ${\sigma }_{AB}$ fulfill the formula (14).
  14 $${\sigma }_{A}>{\sigma }_{B}+{\sigma }_{AB}$$
3. *Partial engulfing* Fig. 2b depicts a Janus structure formed by droplets of phase A and droplets of phase B when the formulas (12), (13), and (14) for ${\sigma }_{A}$, ${\sigma }_{B}$, and ${\sigma }_{AB}$ are not fulfill, the droplets of phase A and phase B share an interface, and both phases are apparent in the continuous phase.

Core/shell microdroplet passage through a continuous phase and the dependence of physical properties, including surface tension can be quantified using a large number of dimensionless groups. Reynolds number ($Re$), Weber number ($We$), and Capillary number ($Ca$) are three of the most well-known examples. $We$, which defines the droplet capability to overcome the interface resistance, and $Ca$, which represents the equilibrium between local shear stresses and capillary pressure, are used to study the various states that can occur during a micro-droplet’s passage and has a substantial impact on the transition between individual flow regimes.

According to the key points in microfluidic devices such as droplet formation, transportation, sorting^53^, and performance, several common liquid–liquid–liquid flow patterns were observed in microchannel (e.g., dripping, jetting, etc.). These flow patterns depend on microchannel geometry, microchannel size, inlet junctions, microchannel wettability, and liquid properties. As mentioned earlier, the formation of different patterns of core/shell microdroplet flow in the microchannel was controlled by the competition between the viscous force and the interfacial tension force. So, two phases (shell and continuous phases) that play a significant role in droplet formation were used to make a model that could predict how flow patterns depend on the velocities and properties of the fluid.

As mentioned, the competition between inertia and capillary pressure is modeled using the Weber number^49^:

15 $$We=\frac{\rho {u}^{2}d}{\sigma }$$

where $\rho$ is the phase density, $u$ is the microdroplet velocity, $\sigma$ is the interfacial tension between the phases, and $d$ is the equivalent diameter of the microdroplet. The $We$ number reflects how significant the kinetic energy of the core/shell micro-droplet is in comparison to the deformation resistance of the interface. Therefore, this number incorporates the properties of both the core and shell liquids. $We$, defines interfacial tension as a measurement of the resistance of an interface to droplet engulfment. According to the micro-droplet diameter range and corresponding velocity changes, the $Re$ number of the droplet, defined as $Re=\rho ud/\mu$ ^49^, is about 50–250. While some of the variables are kept constant, the relative variables are varied for the variation of the mentioned numbers within the standard limits. Along with the core/shell droplet diameter, these values also include the material properties of three phases. The range of the relevant variables used in this investigation is displayed in Table 4.

**Table 4:** Operating value or ranges of variables.

Variables	Notation	Value or range	Unit
Density of the core phase	${\rho }_{core}$	$2299.03$	($\mathrm{kg}/{\mathrm{m}}^{3}$)
Viscosity of the core phase	${\mu }_{core}$	$0.0050$	($\mathrm{Pa s}$)
Density of the shell phase	${\rho }_{shell}$	$2188.37$	($\mathrm{kg}/{\mathrm{m}}^{3}$)
Viscosity of the shell phase	${\mu }_{shell}$	$0.0054$	($\mathrm{Pa s}$)
Density of the continuous phase	${\rho }_{c}$	$917$	($\mathrm{kg}/{\mathrm{m}}^{3}$)
Viscosity of the continuous phase	${\mu }_{c}$	$0.0411$	($\mathrm{Pa s}$)
Interfacial tension between core and shell phases	${\sigma }_{core,shell}$	$0.1\times {10}^{-3}$	($\mathrm{N}/\mathrm{m}$)
Interfacial tension between core and continuous phases	${\sigma }_{core,c}$	$2.2\times {10}^{-3}$	($\mathrm{N}/\mathrm{m}$)
Interfacial tension between shell and continuous phases	${\sigma }_{shell,c}$	$1.8\times {10}^{-3}$	($\mathrm{N}/\mathrm{m}$)
Weber number of the core phase	${We}_{core}$	$1.14\times {10}^{-4}-63.7$	–
Weber number of the shell phase	${We}_{shell}$	$8.46\times {10}^{-5}-30.4$	–
Capillary number of the continuous phase	${Ca}_{c}$	$2.65\times {10}^{-2}-4.12$	–

## Results and discussion

First, droplet evolution and validation in the co-flow microchannel are examined in this section. The CFD results of core/shell droplet formation in the stated configuration are studied in the following stage, and the flow patterns of the droplet formation are discussed. Finally, a phenomenological model for estimation of shell thickness is proposed.

### Validation

**Figure 3:**
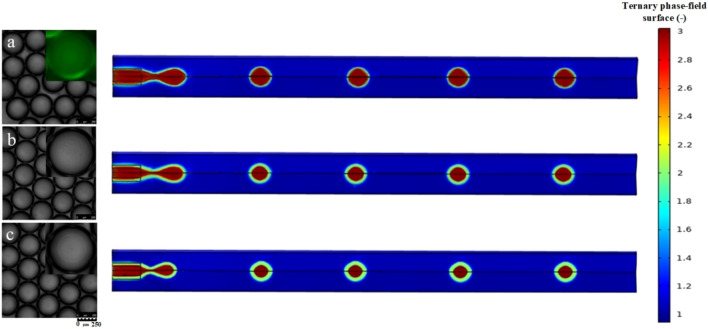
Comparison of the experiment^27^ and numerical simulation for formation of core/shell microdroplets at different flow rate ratios of ${u}_{shell}:{u}_{core}$, (**a**) 1:1, (**b**) 2:1, (**c**) 3:1; The scale bar was 250 µm.

Numerical results were validated using experimental data provided by Li et al.,^27^ to ensure their accuracy. They experimented with the formation of Dextran/silica@Poly (ethylene glycol) diacrylate/silica microdroplets through the soybean oil fatty acid methyl ester as a continuous phase. Due to the phase separation of PEGDA and DEX aqueous solutions, the novel developed microchannel device can easily prepare DEX@PEGDA core–shell microdroplets. Different three constant numbers are used to characterize the interfacial tension between the core/shell, core/continuous, and shell/continuous phases. Using respective material properties and similar conditions as given in Table 4, a numerical simulation was conducted. Figure 3 compares the experimental image sequences with the numerical simulations of the volume fraction contour.

The experimental results depicted in Fig. 3 indicate that DEX/silica@PEGDA/silica core–shell microdroplets with the same size of approximately 300 µm were successfully prepared at varying V_PEGDA_:V_DEX_ flow rate ratios. Microdroplets of uniform sized were produced by a constant total flow rate of aqueous phase and oil phase (continuous phase). However, the DEX/silica and PEGDA/silica flow rate ratios cause the core size and shell thickness to vary. The shell thickness of PEGDA was minimal when the ratio of V_PEGDA_ to V_DEX_ flow rates was 1:1. Figure 3b,c illustrate that as the V_PEGDA_:V_DEX_ ratio rises, the DEX/silica core reduces while the PEGDA/silica shell increases. When the flow rate ratios of V_PEGDA_:V_DEX_ were 1:1, 2:1, and 3:1, respectively, the shell thickness-to-microdroplet diameter ratio was 1:30, 1:7, and 1:5, respectively. The results show that core–shell microdroplets made of DEX/silica@PEGDA/silica may be easily formed in the microchannel device using a variety of core diameters and shell thicknesses.

As illustrated in Fig. 3, the numerical simulation well reproduces the observed concept qualitatively. As an example, both experimental and numerical images show core/shell droplet nucleation and growth, core/shell droplet detachment, and core/shell droplet chain release. The core/shell droplet size predictions were compared to experimental data obtained over the same time intervals for quantitative validation. Figure 3 demonstrates a close agreement between the numerical simulation and the experimental observation. Furthermore, the size of the core/shell droplet following detachment was determined and compared to experimental data at different flow ratios of ${u}_{shell}:{u}_{core}$. The estimated size of the droplet at that time was $309\, \upmu \mathrm{m}$, while the experimentally measured value was $300\, \upmu \mathrm{m}$, indicating a 2.9% relative error. It should be noted that the ratio of core to shell flow rates determines both the core size and the shell thickness. When the flow rate ratio of ${u}_{shell}:{u}_{core}=1:1$, the shell was excessively thin (Fig. 3a). As can be seen in Fig. 3b,c, as the ${u}_{shell}:{u}_{core}$ ratio increases, the shell phase becomes thicker and the core phase shrinks. Based on these results, it appears that forming core/shell microdroplets with a wide range of core sizes and shell thicknesses in a microchannel device is relatively simple.

In general, the formation and movement of a core/shell droplet in a microfluidic medium are three-dimensional. Consequently, the errors were caused by the assumption of axisymmetric in two dimensions. Moreover, errors can divert the measured parameter from the real parameter when evaluating the properties of the fluids, particularly the interfacial tension between three phases (which has a significant effect on the flow pattern). Some inconsistencies in droplet shape and interface deformation were seen due to these inaccuracies, but generally, the numerical model and experimental observations were in good agreement, and the core/shell droplet size and flow pattern were predicted with high accuracy. However, such inaccuracies and discrepancies have no noticeable effect on the flow patterns for the purposes of this investigation. Therefore, the numerical simulation was verified and employed to analyze the flow patterns of core/shell droplet formation in a microfluidics medium through a continuous phase.

### Flow patterns

As mentioned in the literature review, two-phase flow simulation, droplet formation, and two-phase flow map presentation using microfluidics devices have been widely studied. But in the field of three-phase flow simulation and core/shell microdroplet formation, a handful of studies have been performed. So far, no flow map considering three phases has been presented. This research aims to create a complete flow map that takes into account all different patterns using dimensionless numbers for the first time.

The stability of the flow in the microchannel depends linearly on the flow rate ratio of the phases, capillary number of the continuous phase, and microdroplet dynamics. At first, a higher flow rate in the continuous phase presents a higher velocity gradient and a higher pressure on the interface, which indicates a greater viscous shear force and thus promotes core/thread elongation and neck thinning, ultimately resulting in the breakdown of the dispersed core or thread into shorter flows. Secondly, a higher flow rate of the shell phases shows a stronger inertia due to the shell phase being continuously fed by a pump. Consequently, the increased inertia of the shell phase provides a higher resist to the elongation and thinning processes induced by the continuous phase, delaying the breakup and resulting in longer flows.

When a core/shell droplet detaches from a micro-channel tip, various parameters such as fluid physical properties and droplet size might influence the droplet's passage through the continuous phase. Some flow patterns may appear during the flow transition in the micro-channel downstream, depending on the relative values of the effective parameters. By varying the effective parameters, five major flow patterns can be observed: core/shell dripping, core/shell jetting, core/shell slug, tubing, and parallel flows. Each of the flow patterns will be investigated separately in the following sections.

#### Core/shell dripping/slug

Viscous forces that pull the interface to rupture dominate over interfacial tension effects that stabilize the forming droplet against breakage in the dripping mode as the capillary number of the continuous phase increases. Breakup occurs directly at the shell nozzle in the micro-channel in dripping mode. The droplet keeps its spherical shape with a size less than the channel diameter as a result of the high viscous shear force that causes the shell phase fluid to break up before the emerging droplet obstructs micro-channels. When the viscous stress remains constant, uniform droplets are formed. The dripping regime has several advantages, including easy control and remarkable reproducibility.

**Figure 4:**
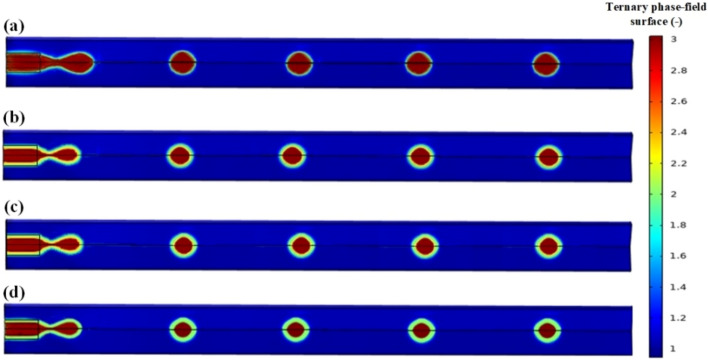
Dripping regimes in the co-flow micro-channel for core/shell microdroplet; Core, shell, and continuous phase’s velocity and shell thickness, respectively: (**a**) 0.00088 m/s, 0.002126 m/s, 0.00655 m/s, and 15.79 µm, (**b**) 0.00088 m/s, 0.003827 m/s, 0.00655 m/s, and 31.16 µm, (**c**) 0.00088 m/s, 0.004678 m/s, 0.00655 m/s, and 47.37 µm, (**d**) 0.00088 m/s, 0.005954 m/s, 0.00655 m/s, and 62.34 µm.

**Figure 5:**
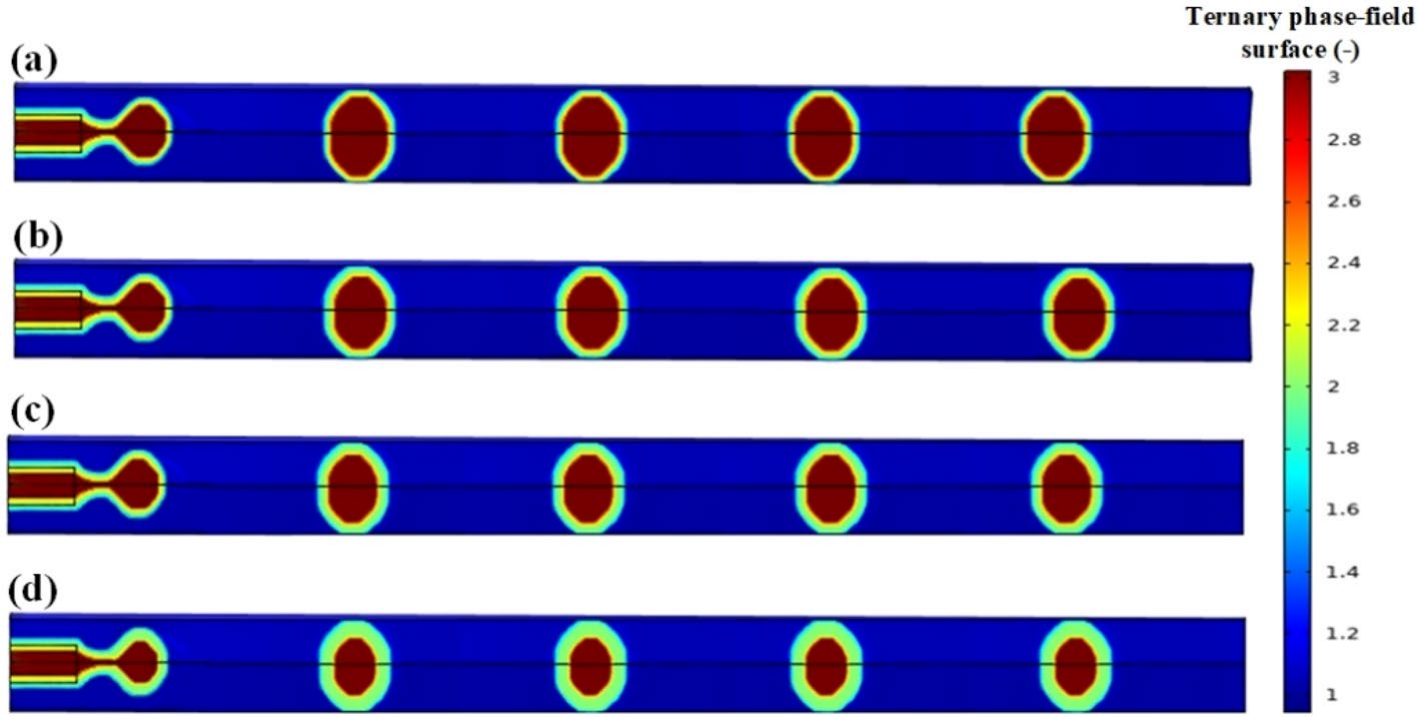
Slug regimes in the co-flow micro-channel for core/shell microdroplet; Core, shell, and continuous phase’s velocity and shell thickness, respectively: (**a**) 0.000288 m/s, 0.000546 m/s, 0.0035 m/s, and 17.65 µm, (**b**) 0.000288 m/s, 0.000682 m/s, 0.0035 m/s, and 34.78 µm, (**c**) 0.000288 m/s, 0.000773 m/s, 0.0035 m/s, and 52.94 µm, (**d**) 0.000288 m/s, 0.00091 m/s, 0.0035 m/s, and 89.55 µm.

The key to creating a droplet or slug flow is dispersing one phase (the shell phase) in the other phase (continuous phase) to form suitable droplets or slugs. In fact, droplet and slug generation mechanisms are quite similar. Droplets, as illustrated in Fig. 4, have diameters lower than the micro-channel depth or width and hence do not undergo any deformation, whereas slugs, which are larger than these dimensions, must undergo deformation in order to fit the micro-channel as shown in Fig. 5. The transition from a dripping regime to a slug flow is typically accomplished by increasing the shell phase flux and decreasing the continuous phase flux. During a slug flow, the interface area between the phases is greatly increased due to the presence of a thin layer of the continuous phase between the wall and each deformed slug. In addition, a vortex flow forms in the upper half of the slug and an anti-vortex flow forms in the lower half as a result of the shear force between the thin film and the slug. Parallel to this, the shear force between the wall and the continuous phase adjacent to the slug induces two internal recirculating vortex flows with opposite recirculating directions. These internal swirling flows are completely chaotic. As illustrated in Figs. 4 and 5, the thickness of the shell increases by increasing the velocity ratio of the shell phase to the core phase at a constant velocity of the carrier phase fluid.

#### Core/shell jetting

**Figure 6:**
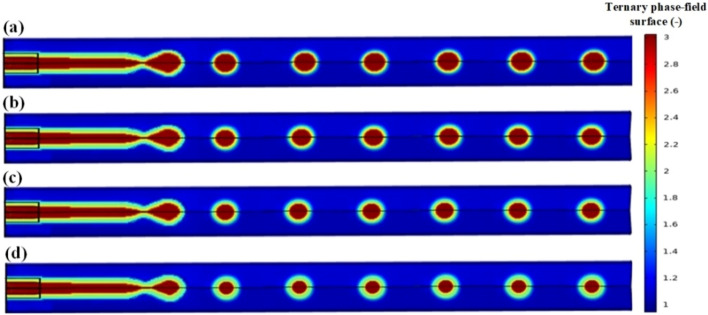
Jetting regime in the co-flow micro-channel for core/shell microdroplet; Core, shell, and continuous phase’s velocity and shell thickness, respectively: (**a**) 0.00729 m/s, 0.0165 m/s, 0.0455 m/s, and 15.58 µm, (**b**) 0.00729 m/s, 0.021 m/s, 0.0455 m/s, and 31.17 µm, (**c**) 0.00729 m/s, 0.03 m/s, 0.0455 m/s, and 38.46 µm, (**d**) 0.00729 m/s, 0.0375 m/s, 0.0455 m/s, and 54.55 µm.

An increase in either the continuous-fluid or shell-fluid flow rate causes a transition from dripping to jetting, in which a long liquid jet emerges from the shell phase channel before breaking up into droplets. The jetting regime is characterized by a stream (jet) of the inner liquid that extends into the outlet channel, droplets that pinch off further downstream, and the tip of the inner phase that persists in the outlet channel following break-up. Droplet formation may occur more frequently (1–2 orders of magnitude) in the jetting regime compared to the dripping regime. Figure 6 shows the jetting regime at different velocities of the three core/shell/carrier phases.

In the jetting regime, more polydisperse droplets are formed when subjected to capillary perturbations than in the dripping regime. When the combined viscous forces of the continuous-fluid and the shell-fluid inertia are higher than the interfacial tension forces, jetting occurs for the co-flowing liquid streams. According to the force balance argument, jets in co-flow geometries can be classified as either narrowing jets or widening jets.

#### Tubing

Within the confines of the tubing regime, the core phase flows continuously within the channel's central region, while the shell phase flows within the annulus formed by the core phase's central region, and the continuous phase also flows in the annulus formed by the shell phase center and the channel walls. At relatively high flow rates of the shell phase, small interfacial waves and undulations can be seen along the interface. However, within the viewing area, there is no distinction along the liquid thread. In most cases, the continuity of the thread can be maintained across a considerable distance.

**Figure 7:**
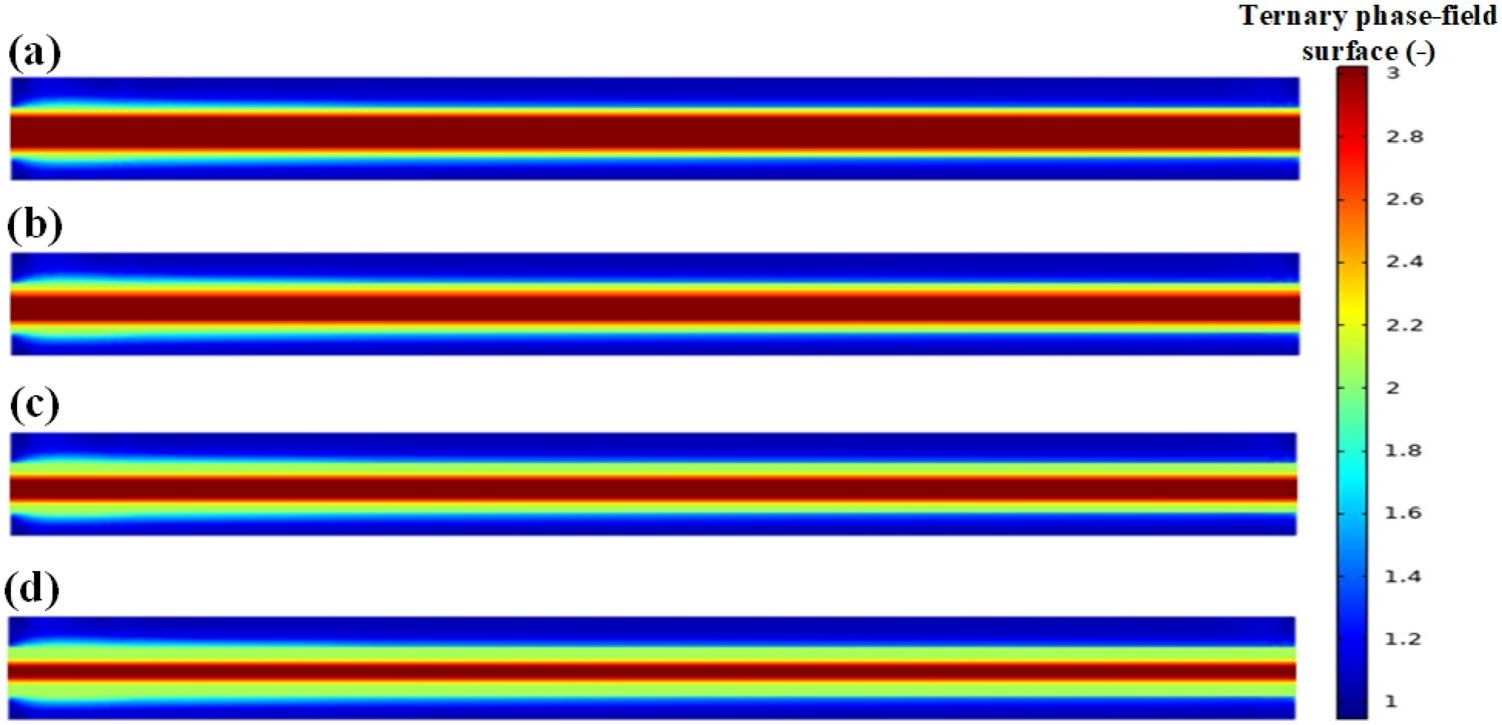
Tubing regime in the co-flow micro-channel for core/shell microdroplet; Core, shell, and continuous phase’s velocity and shell thickness, respectively: (**a**) 0.084 m/s, 0.1076 m/s, 0.0633 m/s, and 18.18 µm, (**b**) 0.084 m/s, 0.1329 m/s, 0.0633 m/s, and 27.27 µm, (**c**) 0.084 m/s, 0.1646 m/s, 0.0633 m/s, and 63.63 µm, (**d**) 0.084 m/s, 0.1899 m/s, 0.0633 m/s, and 76.12 µm.

A viscous core that represents the majority of the main micro-channel's cross section downstream from the junction corresponds to the tubing regime. The tubing regime normally occurs at low continuous phase flow rates. In this regime, three phases flow in the vicinity of each other, with the exception that the high velocity of the phases prevents core/shell phase breaking and core/shell droplet formation. Increasing the velocity of the shell phase, like in the dripping regime, will also result in an increase in shell thickness, as shown in Fig. 7.

#### Laminar/parallel flow

Laminar flow in the co-flow microchannel is also known as parallel flow in the literature. Laminar flow occurs when three immiscible liquids flow continuously and parallel to one another without dispersing. Core/shell phase transfer occurs by molecular diffusion between phases, and a uniform, continuous, and stable interface is created between the three immiscible liquid lamellae. The inlet structure and interface stability are the two most important factors in creating a laminar flow. Important factors affecting the stability of the interface are high flow rates of phases, physical properties, surface modification, guide structure, intermittent partition wall, micro-porous membrane, and surfactant.

**Figure 8:**
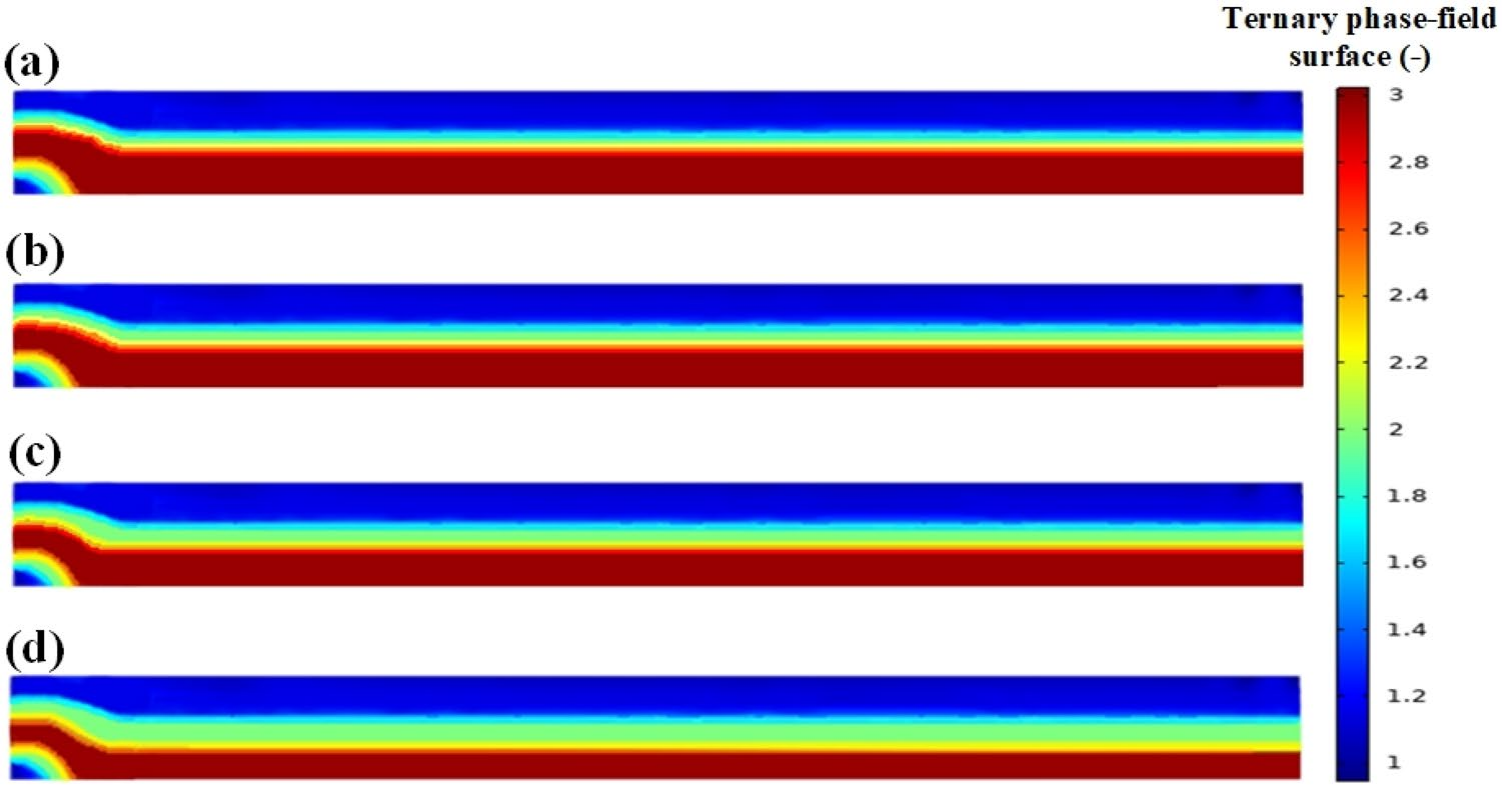
Laminar/parallel regime in the co-flow micro-channel for core/shell microdroplet; Core, shell, and continuous phase’s velocity and shell thickness, respectively: (**a**) 0.0201 m/s, 0.04178 m/s, 0.0882 m/s, and 42.86 µm, (**b**) 0.0201 m/s, 0.05849 m/s, 0.0882 m/s, and 56.67 µm, (**c**) 0.0201 m/s, 0.06684 m/s, 0.0882 m/s, and 85.71 µm, (**d**) 0.0201 m/s, 0.08356 m/s, 0.0882 m/s, and 119 µm.

The difference in the value of the contact angle between this regime and the previous regimes is significant. If the contact angle between the three phases is slightly lowered, the core phase attaches to the wall first, and then the shell phase attaches to the core phase, and the carrier phase fluid attaches to the shell phase and flows along with it. As a result, this regime might be considered distinct from the prior regimes. However, in this study, it was attempted to investigate all five regimes concurrently. In this regime, increasing the velocity of the shell phase causes an increase in shell phase thickness (Fig. 8).

### Flow regime map

**Figure 9:**
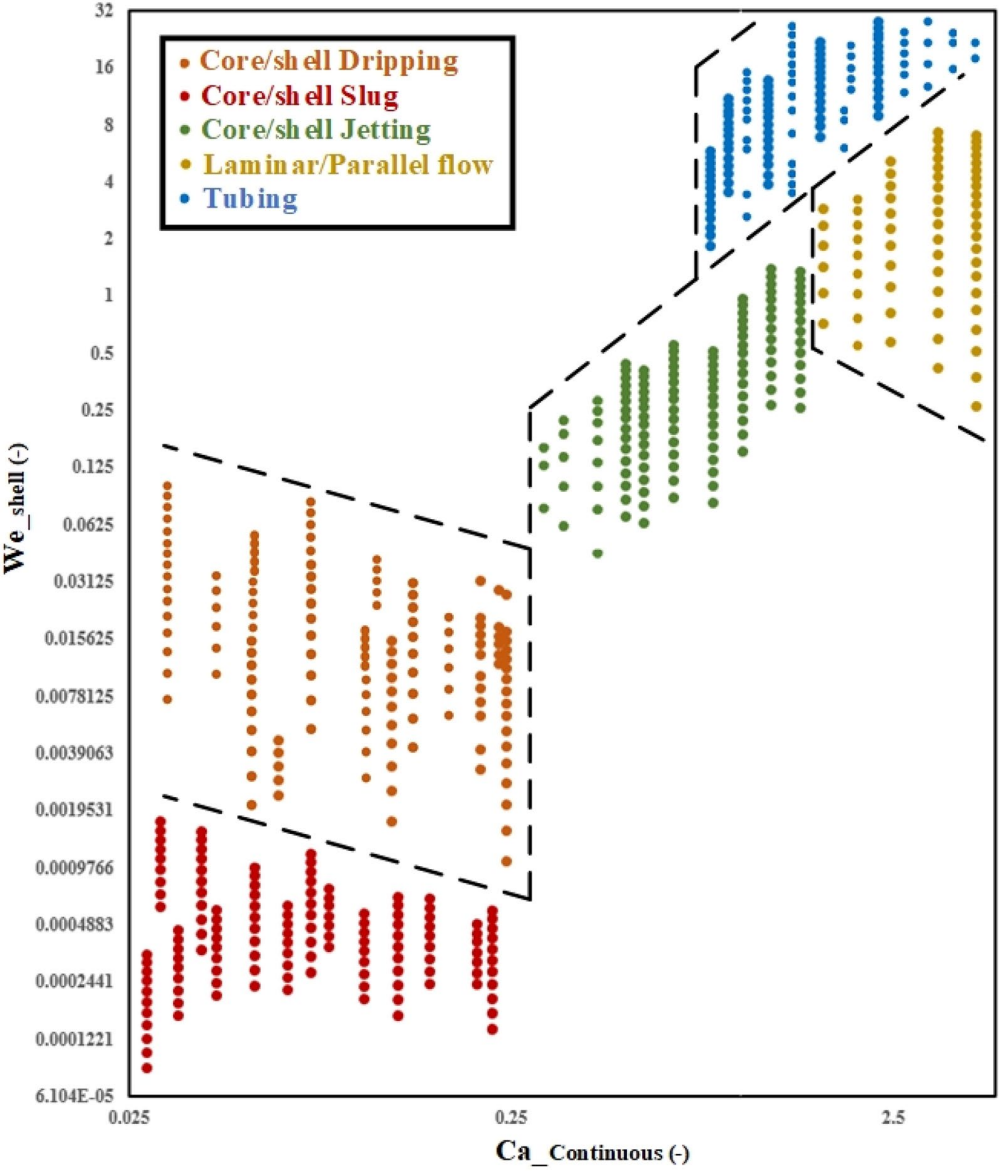
Non-dimensional flow regime map with transition criteria for core/shell microdroplets formation in the co-flow microchannel.

${We}_{shell}$ And ${Ca}_{c}$ were utilized to identify distinct flow regimes across a wide range of physical parameters, as explained above. The flow map depicts five separate regimes: core/shell dripping, slug, jetting, tubing, and laminar/parallel flow based on ${We}_{shell}$ and ${Ca}_{c}$ values. At low ${We}_{shell}$ numbers and low ${Ca}_{c}$ numbers, slug flow can be observed, as illustrated in Fig. 9. A low ${We}_{shell}$ number indicates either low microdroplet kinetic energy or high interfacial tension. Therefore, the droplet does not have the ability to be surrounded by a continuous phase. The small droplet diameter, low density, and high viscosity of the shell phase all contribute to the droplet's slow velocity, which is necessary for low kinetic energy. Since the diameter of the core/shell microdroplet is a power of 4 in the simplified ${We}_{shell}$ relation, it exerts the most control over the ${We}_{shell}$ number. If the ${Ca}_{c}$ number is high, then either the surface tension is low or the viscosity and velocity of the continuous phase are high. For example, the shell phase cannot form as a droplet if the viscosity of the continuous phase is high.

At large ${We}_{shell}$ numbers and large ${Ca}_{c}$ numbers, a flow regime known as tubing flow is generated. For a given ${We}_{shell}$ number, the droplet's kinetic energy is high enough to pull and keep some of the continuous phase. As previously indicated, the microdroplet diameter plays a significant role, and larger droplets have a larger ${We}_{shell}$ number. Thus, given a substantial stream of shell phase, the chance of a tubing regime is high. However, another parameter, the ${Ca}_{c}$ number, is crucial for determining the regime. For the tubing regime, not only is a high ${We}_{shell}$ number necessary, but also a high ${Ca}_{c}$ number.

If the velocity difference between the three phases is insignificant and the velocity of the continuous phase is slightly higher than the other phases, the laminar/parallel flow regime occurs when the three phases are placed next to each other in the microchannel downstream. As a result, in laminar/parallel flow, capillary and Weber numbers are in the same order. In the dripping flow regime, the velocity of the shell phase is low and the velocity of the continuous phase is high, and the inertia of the continuous phase causes the separation of the shell phase, which flows in the form of droplets in the downstream of the channel. This regime occurs in medium capillary and low Weber numbers. Now, if the velocity of the continuous phase is so high that the formed droplets stick together in the three-phase mixing area and a jet length is formed, the flow regime changes to a jet, which has higher capillary numbers than the dripping regime.

Only the ${We}_{shell}$ number is essential for low ${Ca}_{c}$ values (${Ca}_{c}<0.28$), as seen in Fig. 9. When ${We}_{shell}\le 0.00294-0.00823{Ca}_{c}$, the slug flow pattern is seen, and when $0.00294-0.00823{Ca}_{c}\le {We}_{shell}\le 0.20144-0.5576{Ca}_{c}$, the dripping flow pattern is observed. For intermediate ${Ca}_{c}$ numbers ($0.28<{Ca}_{c}<0.97$), the jetting flow regime is observed when ${We}_{shell}\le 4.548{Ca}_{c}-1.022$. The kinetic energy of the microdroplet increases as ${We}_{shell}$ number increases. Furthermore, for quite large ${We}_{shell}$ numbers, the ${Ca}_{c}$ number is the only important parameter. The laminar/parallel flow regime is determined when ${Ca}_{c}>0.97$ and $0.634-0.0915{Ca}_{c}\le {We}_{shell}\le 4.548{Ca}_{c}-1.022$. When ${We}_{shell}$ is high (${We}_{shell}\ge 4.548{Ca}_{c}-1.022$), by increasing ${Ca}_{c}$ values (${Ca}_{c}>0.71$), the tubing flow regime is seen. It should be noted that at moderate values of ${We}_{shell}$ and ${Ca}_{c}$, the flow regime determination is linearly related to both dimensionless numbers.

As seen in Fig. 9, five different flow patterns occur for core/shell microdroplet formation in the microchannel. But there are empty zones between these patterns for the following two reasons:

- In order to comprehensively investigate all different flow patterns, the dimensionless Weber number of the shell phase is varied over a large range. Consequently, the empty area between the patterns increases. Similar to earlier studies for two-phase systems, the majority of them investigated into two patterns of parallel and tubing or two patterns of dripping and slug flow. Therefore, the empty zone in Fig. 9 reaches the lowest value possible if we evaluate the studied patterns in this manner (i.e., a flow map for both patterns). The high velocity of the shell phase contributes to the instability of the jetting regime as well. The jetting regime that occurs in the microchannel is the most stable form in the region depicted in Fig. 9. Changing the boundary conditions (flow rates) of each phase when transitioning patterns helped ensure the study's validity.
- Microchannel geometries vary widely in terms of cross-sectional area, main channel form, and connector configuration for mixing. Both the flow pattern and the core/shell microdroplet size are extremely affected by the geometry of the three-phase junction and the three-phase mixing region. By decreasing the diameter of the microchannel, the specific surface area between shell/continuous phases and surface forces increase while volumetric forces decrease. Consequently, the size of microdroplets reduces. Since in this study only one type of microchannel with a specific size has been investigated, it is expected that instability will occur at some points that were omitted from the investigation.

The use of this flow map in a medical application is possible. Depending on the goal of the formation and development of the core/shell microdroplet in the co-flow microchannel, the appropriate condition can be chosen. The dripping/slug/jetting regime is appropriate for operations like fluid transmission or particle encapsulation. It is possible to transmit one fluid to another through a third fluid (the middle fluid) using this regime and additional considerations. Additional research on this subject is required. The dripping regime is now utilized to improve mass transfer and mixing between two stratified immiscible liquids. The tubing/laminar flow regime can be used to move fluids through a second or third phase without mixing the phases or causing a lot of interface deformation.

This work exclusively focuses on the situations in which core/shell microdroplets can form and flow through the downstream of the co-flow microchannel. In the presence of high ${We}_{shell}$ and ${Ca}_{c}$ numbers, the middle phase is impossible to form the core/shell microdroplet. As a result, the flow map is created for the passage and flow state. The dripping regime can be thoroughly studied and justified in future studies. Furthermore, the flow regimes for the formation of core/shell microdroplets in another microchannel could be an interesting issue for future investigation.

### Correlation between shell thickness and shell-to-core phase velocity ratio

**Figure 10:**
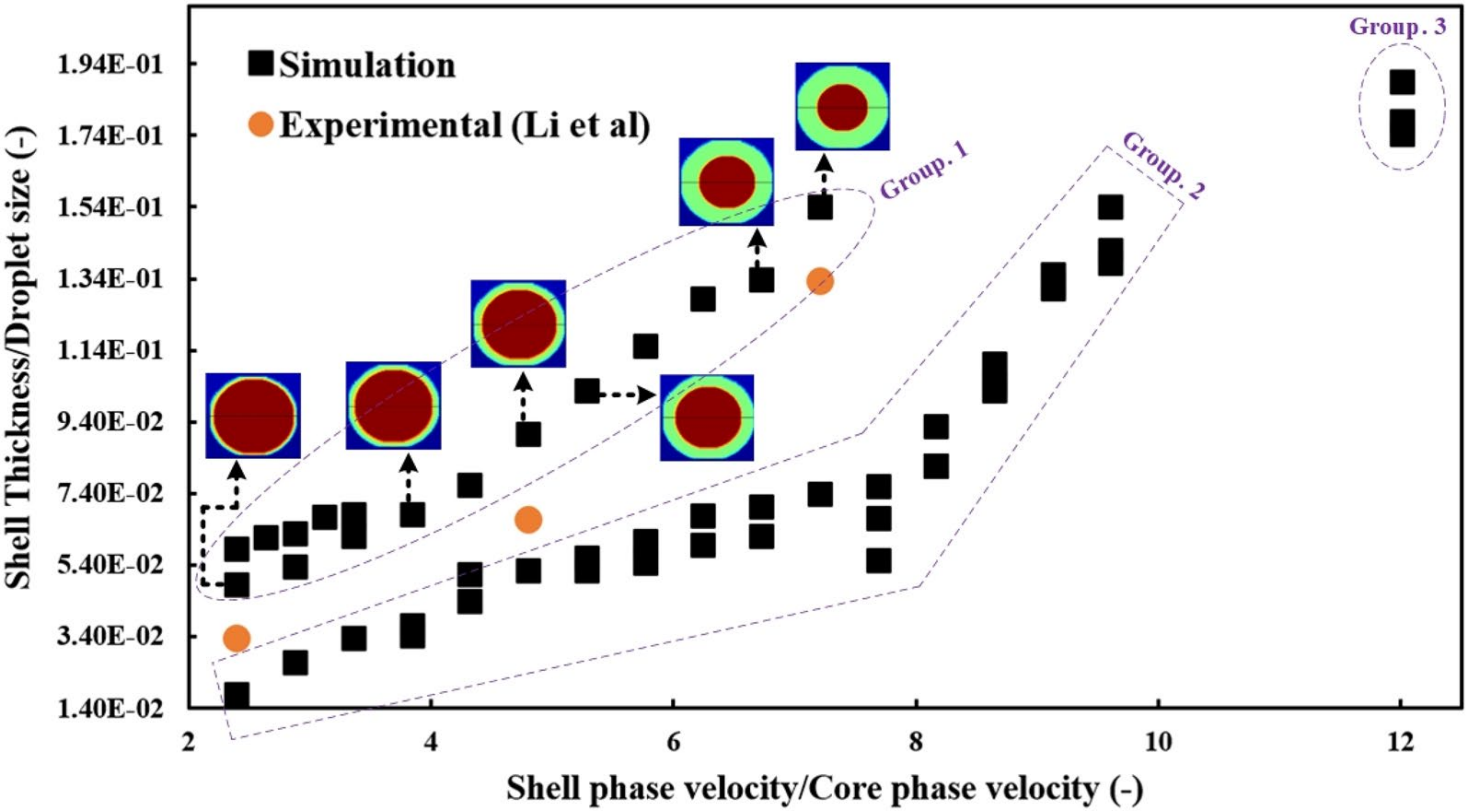
The ratio of shell thickness to microdroplets size in terms of the shell-to-core phase velocity ratio; CFD simulation and Experimental study of Li et al.^27^.

To estimate the shell thickness of the formed droplets in the dripping regime, a correlation can be expressed in terms of the core, shell and continuous phase’s velocity, surface tensions, and physical properties. For this purpose, by performing 51 CFD simulations for different velocities of three phases, droplets with different sizes and shell thicknesses were formed. Figure 10 shows the ratio of shell thickness to microdroplet size in terms of the shell-to-core phase velocity ratio.

There are three groups of points, as shown in Fig. 10, and each group is evaluated at a constant velocity of the core phase. The fluid velocity of the continuous phase is low ($0.00655\, \mathrm{m}/\mathrm{s}$ and $0.0033 \,\mathrm{m}/\mathrm{s}$) in the first group, and the obtained core/shell microdroplet has a diameter of 309 µm and 421 µm, respectively. On the other hand, it is expected that for all points in the first group, the shell thickness will increase as the ratio of the shell-core phase velocity increases. (${u}_{shell}=0.002126\frac{m}{s}-0.005954\frac{m}{s}\to Shell\,thickness=15.7\, \upmu \mathrm{m}-62.3\, \upmu \mathrm{m}$).

In the second group, the continuous phase's velocity increases relatively ($0.011144 \,\mathrm{m}/\mathrm{s}$, $0.012455\, \mathrm{m}/\mathrm{s}$ and $0.01311\, \mathrm{m}/\mathrm{s}$). By applying more inertia to the shell phase fluid, the size of the core/shell microdroplets is reduced to 228 µm, 203 µm and 191 µm, respectively, and in a similar manner, the thickness of the shell also decreases. As a result, it is expected that by increasing the carrier phase fluid's velocity, the points of the second group will be formed lower than the points of the first group. (${u}_{shell}=0.002126\frac{m}{s}-0.008505\frac{m}{s}\to Shell\,thickness=3.4\, \upmu \mathrm{m}-29.4\, \upmu \mathrm{m}$).

The only difference between the third and second groups is that the velocity of the shell phase in these three points is very high (${u}_{shell}=0.0106232 \,\mathrm{m}/\mathrm{s}$), causing the shell thickness to increase significantly. The continuous phase velocities of the first, second, and third points (from the top) of the third group are 0.01311 m/s, 0.012455 m/s, and 0.011144 m/s, respectively, and the diameters of the obtained core/shell microdroplets are 191 µm, 203 µm, and 228 µm, respectively. Because the shell-to-core phase velocity ratio (which is constant) increased, the shell thickness increased more than in the second group with an exponential jump.

Finally, the purpose of this section was to demonstrate that Fig. 10 provides a comprehensive understanding of the effects of core/shell velocity values on core/shell microdroplet size and shell thickness.

## Conclusion

Using the phase-field approach in a co-flow microchannel, the formation of core/shell microdroplets through a continuous third phase has been studied numerically. The model was axisymmetric and seemed to have laminar flow. The experimental data given in the literature has been used to validate the model. The computational fluid dynamics simulations and the experimental results were in good agreement. All possible flow regimes have been identified and classified using a computational method. Core/shell dripping, core/shell jetting, core/shell slug, tubing, and laminar/parallel flow were determined to be the five primary flow regimes. The microdroplet size, density, viscosity, and surface tension of the phases are the most important parameters. The competition between inertia and capillary pressure was described using the $We$ number, while the equilibrium between the local shear stresses and the capillary pressure was described using the $Ca$ number. The five separate flow regimes were divided into distinct zones using these dimensionless numbers to create the flow regime map. Moreover, after conducting 51 CFD simulations, a correlation was proposed for shell thickness estimation. The following are the findings for each flow regime:

- The slug flow regime occurs when both ${We}_{shell}$ and ${Ca}_{c}$ are low. The flow regime is always slug flow for ${Ca}_{c}<0.28$ and ${We}_{shell}\le 0.00294-0.00823{Ca}_{c}$.
- At relatively higher Weber numbers ($0.00294-0.00823{Ca}_{c}\le {We}_{shell}\le 0.20144-0.5576{Ca}_{c}$) and the same capillary number (${Ca}_{c}<0.28$) as compared to the slug regime, the dripping regime occurs. In this regime, the inertia of the continuous phase is high enough that the shell phase is completely surrounded by the continuous phase.
- By increasing the inertia of the continuous phase, the droplets clump together and create a jet length in the three-phase mixing region. At moderate capillary ($0.28<{Ca}_{c}<0.97$) and Weber ${We}_{shell}\le 4.548{Ca}_{c}-1.022$ numbers, the jetting regime is evident.
- The laminar/parallel flow regime is determined when ${Ca}_{c}>0.97$ and $0.634-0.0915{Ca}_{c}\le {We}_{shell}\le 4.548{Ca}_{c}-1.022$.
- The tubing regime happens when both ${We}_{shell}$ and ${Ca}_{c}$ are high. The flow regime is tubing flow for ${We}_{shell}\ge 4.548{Ca}_{c}-1.022$ and ${Ca}_{c}>0.71$.

## Data Availability

Data are available with the permission of [Salman Movahedirad]. The data that support the findings of this study are available from the corresponding author, [Saeed Ghasemzade Bariki], upon reasonable request.

## List of symbols

$$Ca$$: Capillary number
$d$: Core/shell microdroplet size ($\upmu \mathrm{m}$)
${F}_{st}$: Surface tension Force ($\mathrm{kg}/{\mathrm{m}}^{2}\,{\mathrm{s}}^{2}$)
$$g$$: Gravitational acceleration ($$\mathrm{m}/{\mathrm{s}}^{2}$$)
$${M}_{o}$$: Mobility coefficient ($${\mathrm{m}}^{2}/\mathrm{s}$$)
$$P$$: Static pressure ($$\mathrm{Pa}$$)
$$Re$$: Reynolds number
$$t$$: Elapsed time ($$\mathrm{s}$$)
$$u$$: Fluid velocity ($$\mathrm{m}/\mathrm{s}$$)
$$We$$: Weber number

## Greek symbols

$\varepsilon$: Interface's characteristic size ($\upmu \mathrm{m}$)
$$\eta$$: Chemical Potential ($$\mathrm{J}/\mathrm{kg}$$)
$\theta$: Contact angle (degree)
$$\mu$$: Dynamic viscosity ($$\mathrm{Pa\, s}$$)
$$\rho$$: Density ($$\mathrm{kg}/{\mathrm{m}}^{3}$$)
$$\sigma$$: Surface tension ($$\mathrm{N}/\mathrm{m}$$)
$${\Sigma }_{i}$$: Capillary parameter ($$\mathrm{N}/\mathrm{m}$$)
$\phi$: Phase-field variable

## Subscript

$c$: Continuous phase, carrier phase
$$Core$$: Core phase
$i, j, k$: Each phase counter
$$Shell$$: Shell phase
$$w$$: Wetted wall

## References

[1] Magnaudet J, Mercier MJ (2020). Particles, drops, and bubbles moving across sharp interfaces and stratified layers. Annu. Rev. Fluid Mech..

[2] Tan J, Lu Y, Xu J, Luo G (2013). Modeling investigation of mass transfer of gas–liquid–liquid dispersion systems. Sep. Purif. Technol..

[3] Shum HC, Bandyopadhyay A, Bose S, Weitz DA (2009). Double emulsion droplets as microreactors for synthesis of mesoporous hydroxyapatite. Chem. Mater..

[4] Hayes R, Ahmed A, Edge T, Zhang H (2014). Core–shell particles: Preparation, fundamentals and applications in high performance liquid chromatography. J. Chromatogr. A.

[5] Murua A, Portero A, Orive G, Hernández RM, de Castro M, Pedraz JL (2008). Cell microencapsulation technology: Towards clinical application. J. Control. Release.

[6] Kumar, K. S., Kumar, V. B. & Paik, P. Recent advancement in functional core–shell nanoparticles of polymers: synthesis, physical properties, and applications in medical biotechnology. *J. Nanopart.* **2013** (2013).

[7] Chen J, Clay NE, Park N-H, Kong H (2015). Non-spherical particles for targeted drug delivery. Chem. Eng. Sci..

[8] Mahdavi Z, Rezvani H, Moraveji MK (2020). Core–shell nanoparticles used in drug delivery-microfluidics: A review. RSC Adv..

[9] Galogahi FM, Zhu Y, An H, Nguyen N-T (2020). Core-shell microparticles: Generation approaches and applications. J. Sci. Adv. Mater. Devices.

[10] He D, Wang S, Lei L, Hou Z, Shang P, He X, Nie H (2015). Core–shell particles for controllable release of drug. Chem. Eng. Sci..

[11] Pan D, Chen Q, Zhang Y, Li B (2020). Investigation on millimeter-scale W1/O/W2 compound droplets generation in a co-flowing device with one-step structure. J. Ind. Eng. Chem..

[12] Tomeh MA, Zhao X (2020). Recent advances in microfluidics for the preparation of drug and gene delivery systems. Mol. Pharm..

[13] Wang W, Zhang M-J, Chu L-Y (2014). Microfluidic approach for encapsulation via double emulsions. Curr. Opin. Pharmacol..

[14] Zhang H, Tumarkin E, Sullan RMA, Walker GC, Kumacheva E (2007). Exploring microfluidic routes to microgels of biological polymers. Macromol. Rapid Commun..

[15] Kang N, Perron M-È, Prud'Homme RE, Zhang Y, Gaucher G, Leroux J-C (2005). Stereocomplex block copolymer micelles: Core−shell nanostructures with enhanced stability. Nano Lett..

[16] Blackburn WH, Lyon LA (2008). Size-controlled synthesis of monodisperse core/shell nanogels. Colloid Polym. Sci..

[17] Reiss P, Protiere M, Li L (2009). Core/shell semiconductor nanocrystals. Small.

[18] Ali HS, York P, Blagden N (2009). Preparation of hydrocortisone nanosuspension through a bottom-up nanoprecipitation technique using microfluidic reactors. Int. J. Pharm..

[19] Watanabe K, Ohsato H, Kishi H, Okino Y, Kohzu N, Iguchi Y, Okuda T (1998). Solubility of La–Mg and La–Al in BaTiO3. Solid State Ionics.

[20] Luo J, Maye MM, Lou Y, Han L, Hepel M, Zhong CJ (2002). Catalytic activation of core–shell assembled gold nanoparticles as catalyst for methanol electrooxidation. Catal. Today.

[21] Templeton A, Wuel W, Murray RW (2000). Monolayer-protected cluster molecules. Acc. Chem. Res.

[22] Gawande MB, Goswami A, Asefa T, Guo H, Biradar AV, Peng D-L, Zboril R, Varma RS (2015). Core–shell nanoparticles: Synthesis and applications in catalysis and electrocatalysis. Chem. Soc. Rev..

[23] Huang L, Wu K, He X, Yang Z, Ji H (2021). One-Step microfluidic synthesis of spherical and bullet-like alginate microcapsules with a core–shell structure. Colloids Surf. A.

[24] Gong X, Peng S, Wen W, Sheng P, Li W (2009). Design and fabrication of magnetically functionalized core/shell microspheres for smart drug delivery. Adv. Funct. Mater..

[25] Ma J, Wang Y, Liu J (2017). Biomaterials meet microfluidics: from synthesis technologies to biological applications. Micromachines.

[26] Majedi FS, Hasani-Sadrabadi MM, Emami SH, Shokrgozar MA, VanDersarl JJ, Dashtimoghadam E, Bertsch A, Renaud P (2013). Microfluidic assisted self-assembly of chitosan based nanoparticles as drug delivery agents. Lab Chip.

[27] Li J, Zhang F, Jiang L, Yu L, Zhang L (2019). Preparation of silica@ silica core–shell microspheres using an aqueous two-phase system in a novel microchannel device. Langmuir.

[28] Knauer A, Thete A, Li S, Romanus H, Csaki A, Fritzsche W, Köhler J (2011). Au/Ag/Au double shell nanoparticles with narrow size distribution obtained by continuous micro segmented flow synthesis. Chem. Eng. J..

[29] Van Nguyen H, Kim KY, Nam H, Lee SY, Yu T, Seo TS (2020). Centrifugal microfluidic device for the high-throughput synthesis of Pd@ AuPt core–shell nanoparticles to evaluate the performance of hydrogen peroxide generation. Lab Chip.

[30] Sun J, Zhang L, Wang J, Feng Q, Liu D, Yin Q, Xu D, Wei Y, Ding B, Shi X (2015). Tunable rigidity of (polymeric core)–(lipid shell) nanoparticles for regulated cellular uptake. Adv. Mater..

[31] Costa C, Liu Z, Martins JP, Correia A, Figueiredo P, Rahikkala A, Li W, Seitsonen J, Ruokolainen J, Hirvonen S-P (2020). All-in-one microfluidic assembly of insulin-loaded pH-responsive nano-in-microparticles for oral insulin delivery. Biomater. Sci..

[32] Panday R, Poudel AJ, Li X, Adhikari M, Ullah MW, Yang G (2018). Amphiphilic core–shell nanoparticles: Synthesis, biophysical properties, and applications. Colloids Surf. B.

[33] Liu C, Feng Q, Sun J (2019). Lipid nanovesicles by microfluidics: Manipulation, synthesis, and drug delivery. Adv. Mater..

[34] Ghobashy MM, Mousaa I, El-Sayyad GS (2021). Radiation synthesis of urea/hydrogel core shells coated with three different natural oils via a layer-by-layer approach: An investigation of their slow release and effects on plant growth-promoting rhizobacteria. Prog. Org. Coat..

[35] Angelo LM, França D, Faez R (2021). Biodegradation and viability of chitosan-based microencapsulated fertilizers. Carbohydr. Polym..

[36] Dimkpa CO, Campos MG, Fugice J, Glass K, Ozcan A, Huang Z, Singh U, Santra S (2022). Synthesis and characterization of novel dual-capped Zn–urea nanofertilizers and application in nutrient delivery in wheat. Environ. Sci. Adv..

[37] He Y, Wu Z, Tu L, Han Y, Zhang G, Li C (2015). Encapsulation and characterization of slow-release microbial fertilizer from the composites of bentonite and alginate. Appl. Clay Sci..

[38] de Carvalho Arjona J, das Graças Silva-Valenzuela M, Wang S-H, Valenzuela-Diaz FR (2021). Biodegradable nanocomposite microcapsules for controlled release of urea. Polymers.

[39] Navarro-Guajardo N, García-Carrillo EM, Espinoza-González C, Téllez-Zablah R, Dávila-Hernández F, Romero-García J, Ledezma-Pérez A, Mercado-Silva JA, Torres CAP, Pariona N (2018). Candelilla wax as natural slow-release matrix for fertilizers encapsulated by spray chilling. J. Renew. Mater..

[40] Fang W, Yang J, Gong J, Zheng N (2012). Photo-and pH-triggered release of anticancer drugs from mesoporous silica-coated Pd@ Ag nanoparticles. Adv. Funct. Mater..

[41] Chen C, Gao C, Liu M, Lǚ S, Yu C, Ma S, Wang J, Cui G (2013). Preparation and characterization of OSA/CS core–shell microgel: In vitro drug release and degradation properties. J. Biomater. Sci. Polym. Ed..

[42] Wu W, Zhou T, Berliner A, Banerjee P, Zhou S (2010). Smart core−shell hybrid nanogels with Ag nanoparticle core for cancer cell imaging and gel shell for pH-regulated drug delivery. Chem. Mater..

[43] Mamaeva V, Rosenholm JM, Bate-Eya LT, Bergman L, Peuhu E, Duchanoy A, Fortelius LE, Landor S, Toivola DM, Lindén M (2011). Mesoporous silica nanoparticles as drug delivery systems for targeted inhibition of Notch signaling in cancer. Mol. Ther..

[44] Sahiner N, Ilgin P (2010). Synthesis and characterization of soft polymeric nanoparticles and composites with tunable properties. J. Polym. Sci. Part A Polym. Chem..

[45] Biswas KG, Patra R, Das G, Ray S, Basu JK (2015). Effect of flow orientation on liquid–liquid slug flow in a capillary tube. Chem. Eng. J..

[46] Chinaud M, Roumpea E-P, Angeli P (2015). Studies of plug formation in microchannel liquid–liquid flows using advanced particle image velocimetry techniques. Exp. Therm. Fluid Sci..

[47] Plouffe P, Roberge DM, Macchi A (2016). Liquid–liquid flow regimes and mass transfer in various micro-reactors. Chem. Eng. J..

[48] Tsaoulidis D, Angeli P (2016). Effect of channel size on liquid-liquid plug flow in small channels. AIChE J..

[49] Wu Z, Cao Z, Sundén B (2017). Liquid–liquid flow patterns and slug hydrodynamics in square microchannels of cross-shaped junctions. Chem. Eng. Sci..

[50] Nunes J, Tsai S, Wan J, Stone HA (2013). Dripping and jetting in microfluidic multiphase flows applied to particle and fibre synthesis. J. Phys. D Appl. Phys..

[51] Fu T, Wu Y, Ma Y, Li HZ (2012). Droplet formation and breakup dynamics in microfluidic flow-focusing devices: From dripping to jetting. Chem. Eng. Sci..

[52] Kashid MN, Agar DW (2007). Hydrodynamics of liquid–liquid slug flow capillary microreactor: Flow regimes, slug size and pressure drop. Chem. Eng. J..

[53] Yagodnitsyna AA, Kovalev AV, Bilsky AV (2016). Flow patterns of immiscible liquid-liquid flow in a rectangular microchannel with T-junction. Chem. Eng. J..

[54] Cubaud T, Mason TG (2008). Capillary threads and viscous droplets in square microchannels. Phys. Fluids.

[55] Dessimoz A-L, Raspail P, Berguerand C, Kiwi-Minsker L (2010). Quantitative criteria to define flow patterns in micro-capillaries. Chem. Eng. J..

[56] Filimonov R, Wu Z, Sundén B (2021). Toward computationally effective modeling and simulation of droplet formation in microchannel junctions. Chem. Eng. Res. Des..

[57] Jafari R, Okutucu-Özyurt T (2016). Numerical simulation of flow boiling from an artificial cavity in a microchannel. Int. J. Heat Mass Transf..

[58] Jamalabadi MYA, DaqiqShirazi M, Kosar A, Shadloo MS (2017). Effect of injection angle, density ratio, and viscosity on droplet formation in a microfluidic T-junction. Theor. Appl. Mech. Lett..

[59] Soroor M, Targhi MZ, Tabatabaei SA (2021). Numerical and experimental investigation of a flow focusing droplet-based microfluidic device. Eur. J. Mech. B Fluids.

[60] Wang D, Abbas Z, Lu L, Zhao X, Xu P, Zhao K, Yin P, Liang J (2022). Numerical modeling and analysis of coaxial electrohydrodynamic jet printing. Sci. Rep..

[61] Farhadi J, Sattari A, Hanafizadeh P (2022). Passage of a rising bubble through a liquid-liquid interface: A flow map for different regimes. Can. J. Chem. Eng..

[62] Boyer F, Lapuerta C, Minjeaud S, Piar B, Quintard M (2010). Cahn–Hilliard/Navier–Stokes model for the simulation of three-phase flows. Transp. Porous Media.

[63] Peng X, Wang X, Du Z, Zeng F (2021). Phase-field simulations of precursor film in microcapillary imbibition for liquid–liquid systems. Int. J. Multiph. Flow.

[64] He Q, Kasagi N (2008). Phase-field simulation of small capillary-number two-phase flow in a microtube. Fluid Dyn. Res..

